# Association of Umbilical Cord Perilipin 2 Levels with Neonatal Anthropometric Measurements in Infants of Diabetic Mothers

**DOI:** 10.3390/children11070771

**Published:** 2024-06-25

**Authors:** Kiymet Celik, Nurten Ozkan Zarif, Ikbal Ozen Kucukcetin, Sema Arayici, Zeynep Kihtir, Hale Unver Tuhan, Hakan Ongun

**Affiliations:** 1 Department Neonatology, Akdeniz University, 07070 Antalya, Turkey; nurtenozkanzarif@akdeniz.edu.tr (N.O.Z.); semaarayici@akdeniz.edu.tr (S.A.); zeynepkihtir@gmail.com (Z.K.); hakanongun@akdeniz.edu.tr (H.O.); 2 Medical Biochemistry Laboratory, Akdeniz University, 07070 Antalya, Turkey; ikbalozen@akdeniz.edu.tr; 3 Department of Pediatric Endocrinology, Akdeniz University, 07070 Antalya, Turkey; haletuhan@akdeniz.edu.tr

**Keywords:** perilipin 2, infant of diabetic mother, birth weight, leptin

## Abstract

Background: Perilipin 2 (PLIN2) is a protein that contributes to the formation and stability of lipid droplets. It has been associated with the development of several diseases, particularly related to glucose and lipid metabolism. In infants of diabetic mother (IDM), fetal hyperinsulinaemia leads to increased adipose tissue and macrosomia. The aim of this study was to investigate the relationship between PLIN2 levels and anthropometric measurements in the IDM and to investigate the relationship between PLIN2 levels and IGF-1, IGF-2 and leptin levels. Methods: The study group consisted of IDMs, while the control group consisted of infants born to non-diabetic mother, matched for gestational week and gender. Cord blood samples were collected from all patients to determine PLIN2, IGF-1, IGF-2 and leptin levels. Anthropometric measurements were taken for all patients at birth. Results: There were no differences between the groups in birth weight, birth length, head circumference and body mass index (BMI), but middle arm circumference, triceps, biceps, subscapular and suprailiac skinfold thickness were significantly higher in the IDM. While PLIN2, IGF-1, IGF-2 and leptin levels were similar between groups, there was a strong correlation between PLIN2 levels and IGF-2 and leptin levels. Conclusions: Even if IDMs were not macrosomic, the presence of high subcutaneous adipose tissue was not associated with PLIN2.

## 1. Introduction

Gestational diabetes mellitus (GDM), defined as varying degrees of glucose intolerance that begins or is first recognised during pregnancy. If untreated, GDM leads to increased complication rates both, in the mother and the fetus. Infants of diabetic mothers (IDM) have been observed to experience certain conditions during their development in utero, such as fetal hyperglycaemia, fetal hyperinsulinaemia and increased adipose tissue. These conditions may result in perinatal complications, including macrosomia, hypoglycemia, and perinatal asphyxia in the early stages, as well as an elevated risk of hypertension, obesity, diabetes mellitus, and cardiovascular diseases in the long term [1,2]. Several adipokine, growth factor and hormone levels have been extensively studied regarding the mechanisms that lead to these pathologies in the IDM [3,4,5].

Insulin-like growth factors and their binding proteins influence maternal metabolism. Furthermore, they act as endocrine signals, enhancing placental function and fetal growth [6,7]. The levels of insulin-like growth factor-1 (IGF-1) were found to be associated with an increased risk of macrosomia in the presence or absence of maternal diabetes [8,9].

Leptin is a hormone synthesised by adipocytes that has been shown to have effects on metabolism and food intake. A positive correlation between leptin levels and weight gain in early infancy has been shown in research [10]. Research suggests that cord blood levels are higher in children with GDM and in macrosomic children without GDM, and that these levels are associated with anthropometric measurments [11,12]. In another study, there was evidence that umbilical cord leptin levels have a positive correlation with newborn abdominal adipose tissue and neonatal fat body mass [13,14].

Perilipin 2 (PLIN2) is a member of the perilipin protein family, which is located on the surface of lipid droplets and is involved in their metabolism. Its primary function is to facilitate the formation and maintenance of lipid droplets [15,16]. Perilipin 2 is a protein that has been the subject of recent studies in animals and adults. It has been implicated in the development of many diseases, including disorders of glucose and lipid metabolism [17]. Preclinical studies have shown that elevated levels of PLIN2 are associated with several metabolic disorders, including obesity, diabetes, fatty liver disease, atherosclerosis and cardiovascular disease, and inhibiting PLIN2 has been shown to prevent or alleviate these conditions [18]. Although PLIN2 is primarily a cellular protein, it has also been detected in the circulation and is present in large amounts in both plasma and urine [15].

Macrosomic infants and IDM have been shown to have higher levels of IGF-1, IGF-2 and leptin [11,12,19]. However, there are no data on PLIN2 levels in IDM. In this study, we aimed to investigate the effect of PLIN2 level on anthropometric measurements and the relationship between PLIN2 and IGF-1, IGF-2 and leptin levels in cord blood of IDM in comparison with the control group.

## 2. Material Methods

This cross-sectional, controlled study was conducted at the Akdeniz University Faculty of Medicine, between 20 February 2022 and 20 February 2023. Maternal diagnosis of gestational diabetes was made by an oral glucose tolerance test (OGTT) between 24 and 28 weeks of pregnancy, and their infants were included in this study. The diagnostic criteria for gestational diabetes were defined according to the guidelines of the International Diabetic Pregnancy Study Group [20]. The study protocol was approved by the Ethics Committee of Akdeniz University, and written informed consent was obtained from the parents of the infants. (report number: AU KAEK 2022/385).

### 2.1. Inclusion Criteria

The study group included singleton healty infants born as a single baby after spontaneous and healthy pregnancy from a mother with gestational diabetes mellitus. The control group included babies born as a single infant to mothers who underwent OGTT screening during pregnancy to exclude gestational diabetes. The control group included patients with the same gestational age and gender as the study group, ensuring a match between the two groups.

### 2.2. Exclusion Criteria

Mothers with other pregnancy complications (hypertensive disorders, chorioamnionitis, multiple gestation, emergency caesarean, abnormal fetal presentation), pregestational diabetes mellitus, and newborns with perinatal asphyxia, suspected fetal distress, neonatal resuscitation were excluded.

### 2.3. Data Collection

The body weight of all mothers was measured at birth, and their body mass index (BMI, kg/m^2^) was calculated. Anthropometric newborn measurements (weight, length, BMI, head circumference, left arm circumference) were taken upon birth. Body weight was measured using an electronic weighing machine with a sensitivity of 5 g. The infant’s height was measured with the infant in the supine position, and head circumference was measured using an inelastic tape measure. The tape was placed over the most prominent point at the back of the head, laterally over the parietal region, and anteriorly over the glabella. Fenton growth charts were used to calculate birth z-scores electronically. Anthropometric measurements were taken using standardised measuring equipment (weight scales, height gauges, calipers). Skinfold thickness was measured on the first day of neonatal life using a Harpenden skinfold caliper (Holtain Ltd., London, UK) with a calibration dowel for subscapular, suprailiac, triceps and biceps.

Cardiac structure and function parameters were assessed by two-dimensional pulsed Doppler M echocardiography within the first 72 h of life by a paediatric cardiologist. Intraventricular septum (IVS) was considered hypertrophic if IVS thickness was >6 mm at end diastole.

Blood samples from the umbilical vein were collected for perilipin-2, IGF-1, IGF-2 and leptin by puncture of the umbilical vein immediately after birth but before the placenta was removed. The blood samples were collected in 10 mL gel separator tubes and centrifuged at 4000 rpm for 10 min. The serum portion was then separated and stored at minus 80 °C until the studies were conducted.

### 2.4. Blood Sample Analyses

Serum Perilipin 2, Leptin, and IGF-2 levels (ng/mL) were measured using the SunRed (Shanghai Sunred Biological Technology Co., Shangai, China) ELISA kit. Absorbance values were read at 450 nm on the CA-2000 Micro-plate Reader (CIOM Medical Co., Ltd., China) Elisa Reader. The Concentration Absorbance Graph was used to calculate the levels based on the absorbance values obtained from the standards and their corresponding concentration values.

IGF-1 levels (μg/L) were measured using the chemiluminescent method in the Siemens Immulite 2000 XPi Immunassay System autoanalyzer (Siemens Healthcare Diagnostics Products Ltd., Gwynedd, UK).

### 2.5. Sample Size and Statistical Analyses

We tested the correlation coefficient, which is predicted to be 0.65 for power calculations between perilipin-2 and the measured values. The α level is 0.05 and the power is 90% for a total sample size of 42 patients. Statistical analyses were performed with the SPSS version 26 software package (IBM, Armonk, NY, USA). Numeric data were presented as mean and standard deviation for normally distributed data and as median and interquartile range for non-normally distributed data. Group differences for normally distributed continuous variables were tested using Student’s *t*-test. Differences for non-normally distributed continuous variables were tested using the Mann-Whitney U-test. Relationships between quantitative variables were examined using Pearson and Spearman correlation analyses. Bland-Altman analysis was used to assess the correlation between data (Evans JD. (1996). Straightforward Statistics for the Behavioural Sciences, Thomson Brooks/Cole Publishing Co., Pacific Grove, CA, USA). *p* < 0.05 was considered statistically significant.

## 3. Results

The study included 42 infants of diabetic mothers and 42 control infants. The mean gestational week was 37.5 ± 1.1 weeks, and the mean birth weight was 3.268 ± 498 g. Of the patients, 46.4% (n: 39) were male. There were differences in anthropometric measurements between the groups matched with the same gestational week and sex. Although IDM had a higher birth weight, this difference was not significant. The IDM group exhibited higher upper arm circumference and skin thickness in the triceps, biceps, subscapular, and suprailiac sites (*p* < 0.001 for all except suprailiac, *p*: 0.048) (Table 1).

Mothers with GDM had significantly higher pre-pregnancy weight, weight at birth, and BMI compared to those without GDM (*p* < 0.001, *p* < 0.001, *p*: 0.001) (Table 1). The mean HBA1c levels of mothers with gestational diabetes were 5.5 + 0.74%. Of the mothers with GDM, 45% (n: 19) were treated with insulin, while the remaining were diet controlled.

The median cord PLIN2 level was 7.1 (5.3–11.8) ng/mL in IDMs and 7.2 (5.0–13.6) ng/mL in all patients. Cord PLIN2, IGF-1, IGF-2 and leptin levels were not significantly different between groups (Table 2).

Interventricular septum hypertrophy was found in 19% (n: 8) of the infants born to mothers with GDM. In those with IVS hypertrophy, the mean IVS diameter was 6.35 ± 0.72 mm. There was no correlation between IVS diameter and PLIN2, IGF-1, IGF-2 and leptin levels.

**Figure 1:**
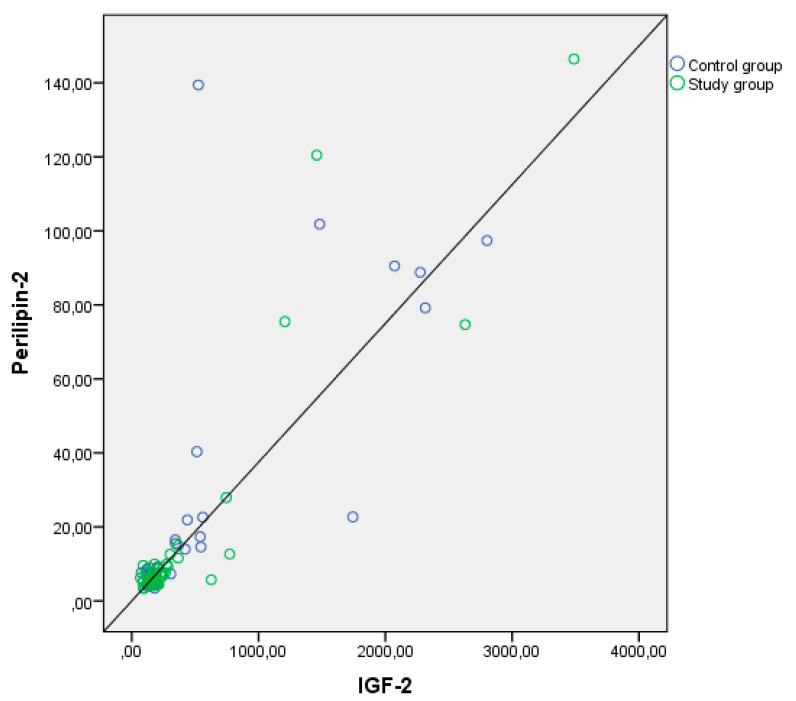
Positive correlation curve between levels of Perilipin-2 and IGF-2 in the cord blood of infants.

**Figure 2:**
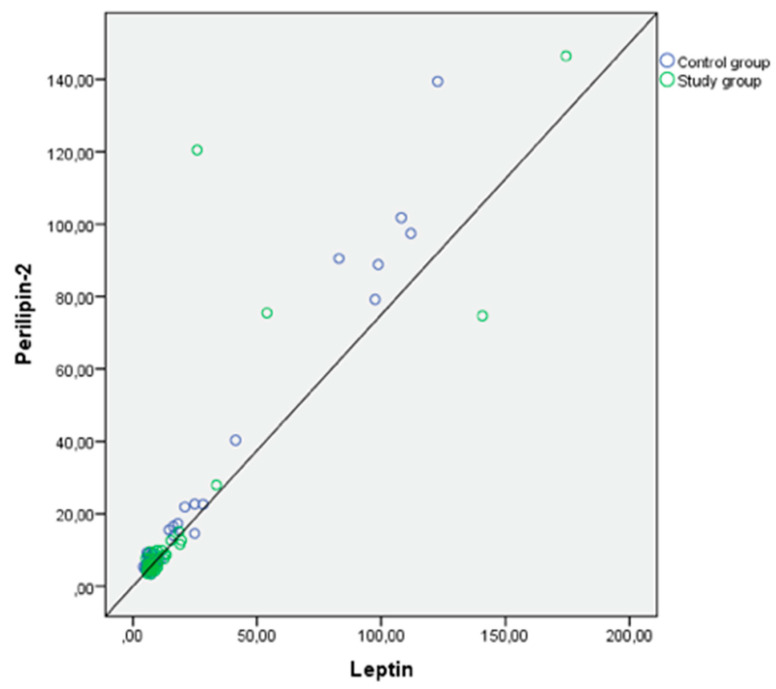
Positive correlation curve between levels of Perilipin-2 and Leptin in the cord blood of infants.

Examining the correlation between anthropometric measurements and cord levels of PLIN2, IGF-1, IGF-2, and leptin, a weak correlation was found between birth weight and PLIN2 levels, and a moderate correlation between birth weight and IGF-1 levels. Additionally, strong positive correlations were found between PLIN2 and leptin, as well as IGF-2 levels (*p* < 0.001, *p* < 0.001) (Table 3). The correlation curve between perilipin-2 and leptin and IGF-2 is shown in Figure 1 and Figure 2.

## 4. Discussion

This study is the first to investigate perilipin-2 blood levels in newborns. The aim of the study was to measure the umbilical cord levels of PLIN2 in the infants of diabetic mothers and to evaluate their relationship with the newborns’ anthropometric indices by comparing them with the healthy group. While there were no differences between the groups in umbilical cord PLIN2, IGF-1, IGF-2, leptin levels and birth weight, birth length and birth head circumference, upper arm circumference, and skinfold thickness were found to be significantly higher in the infants of diabetic mothers. Although pre-pregnancy weight, birth weight, and BMI are significantly higher in mothers with gestational diabetes, the fact that there is no difference in the infants’ birth weight may be well controlled, but the persistence of the difference in other anthropometric measures suggests that other underlying mechanisms are at work. The strong correlation between IGF-2 and leptin levels, which are associated with fetal growth, with PLIN2 levels also suggests that the effect of perilipin-2 on fetal growth cannot be excluded.

Studies on IDM have shown that anthropometric measurements and total body fat are higher than in the healthy group [21,22]. However, some studies have shown that birth weight and other anthropometric measures are similar to control groups, which is associated with well-controlled GDM [3,23,24]. A recent study showing that maternal pre-pregnancy BMI and the presence of GDM had an effect on anthropometric measures showed that the effect of last trimester glucose levels on fetal growth persisted even after correction for maternal anthropometry [25]. In a study comparing infants born to obese, GDM and normal weight mothers, it was found that infants born to maternal obesity and GDM had a higher birth weight than infants born to normal weight mothers. The study found a correlation between maternal weight at the beginning of pregnancy and birth weight in both obese and normal weight mothers, but no such correlation was found in mothers with GDM [22]. The multicenter HAPO study reported that both GDM and maternal obesity have an impact on birth weight, and the risk is even higher when both are present [26]. In this study, it was found that mothers in the IDM group had significantly higher pre-pregnancy and, birth time weight and BMI compared to the control group. However, there was no statistically significant difference between the groups in terms of birth weights, birth length, birth head circumference, and BMI of the patients who were investigated by matching birth week and gender.

In studies, IDMs were evaluated using various measurements in addition to birth weight, birth height and head circumference. A comparison was made between IDM and a control group for chest circumference, abdominal circumference, biacromial distance, thigh circumference, femur length and body proportions. The study found no significant difference between the two groups [24]. Studies examining skinfold thickness, which is considered an important reflection of increased body fat percentage in IDM, have shown varying results. A meta-analysis of 35 studies concluded that IDM increased total body fat amount, triceps, and subscapular skinfold thickness [27]. In a separate study that evaluated IDM and a control group, it was found that triceps skinfold thickness and subscapular skinfold thickness were significantly higher [3]. In a study comparing infants with old GDM and new GDM diagnoses with the healthy group following the revision of the diagnostic criteria for GDM, it was found that while skinfold thickness was high in the old GDM group, skinfold thickness was lower in the new group than in the control group. These results suggest better control [28]. A study investigating the impact of diabetes treatment on skinfold thickness found that skinfold thickness decreased in parallel with the reduction in fat tissue in those who received medical treatment [29,30]. In current study, although there were no differences in body weight and other measurements, skinfold thickness was significantly higher than that of the control group. Differences in GDM diagnosis and management between centres may have contributed to the variations observed in this study and in previous studies.

PLIN2, also known as fat differentiation-associated protein or adipophilin, is a protein involved in intracellular processes such as lipid metabolism, transport and the formation and stability of lipid droplets (LD). It is a marker of LD accumulation and is found on the surface of LDs [31,32]. PLIN2 is expressed in various non-adipose tissues and plays a role in body weight and lipid metabolism by regulating lipid storage and accumulation and preventing lipolysis in these tissues [18,33]. Preclinical and some clinical studies have shown a relationship between PLIN2 overexpression and obesity, atherosclerosis, and Type 2 DM [34]. The number of studies on PLIN in childhood is relatively limited. Among these studies, Tokgöz et al. compared obese adolescent children with healthy children in terms of PLIN polymorphism. Their findings indicated that PLIN6 polymorphism may increase the risk of obesity. Similarly, PLIN1 polymorphism has also been shown to lead to an increased risk of obesity [35], As the patients in these studies were in the child population and genetic polymorphisms were investigated, it is difficult to compare the data with this study. A study demonstrating metabolic alterations in childhood for PLIN2, whose impact on pancreatic β cells is more clearly defined, could not be identified in the literature [36].

This study found a strong correlation between PLIN2 and leptin levels. Previous studies examining PLIN2 and leptin levels have also found a strong correlation [15,37], although the age range and pathologies of the groups studied varied.

It is important to note that our study has several limitations. One limitation of this study is that PLIN2 levels, which are not known during pregnancy, are only cross-sectionally measured in cord blood at birth. Additionally, the small number of macrosomic infants prevents comparison with both macrosomic and normal weight infants.

The results shows no correlation between perilipin 2 and adiposity markers among the studied infants of diabetic mothers. This may be related to the low number of macrosomic infants, the lack of difference in birth weight between the groups, and the well-controlled status of the diabetic mothers. Despite the mothers’ well-controlled gestational diabetes, their infants continue to show an increase in subcutaneous adiposity. This suggests that there may be different underlying pathways. Furthermore, the strong positive correlation between PLIN2 levels in cord blood and IGF-2 and leptin levels supports previous similar studies.

## Figures and Tables

**Table 1:** Comparison of anthropometric measurements of groups.

	Total (n: 84)	İnfants of Diabetic Mothers (n: 42)	Control Group Infants (n: 42)	*p*
Birth weight (gr)	3268 ± 498	3332 ± 531	3205 ± 459	0.24
Birth weight Z-score	0.43 (−0.08 to 1.0)	0.5 (−0.08 to 1.4)	0.39 (−0.1 to 0.39)	0.32
Birth height (cm)	48.8 ± 2	49 ± 2.0	48.6 ± 2.0	0.48
Birth height Z-score	0.17 (−0.23 to 0.59)	0.28 (−0.23 to 0.72)	0.17 (−0.5 to 0.57)	0.23
Head circumference (cm)	34.4 ± 1.2	34.6 ± 1.3	34.3 ± 1.1	0.37
Head circumference Z-score	0.72 (0.08 to 1.13)	1 (0.04 to 1.4)	0.48 (0.17 to 0.81)	0.12
Body mass index (kg/m^2^)	13.6 ± 1.4	13.8 ± 1.5	13.4 ± 1.3	0.28
Arm circumference (cm)	10.0 ± 1.6	10.7 ± 1.6	9.3 ± 1.2	<0.001
Triceps skinfold thickness (mm)	6.5 ± 2.5	7.2 ± 2.3	5.7 ± 2.6	0.006
Biceps skinfold thickness (mm)	6 (4–8)	6.5 (5.5–8)	5 (4–6)	0.001
Subscapular skinfold thickness (mm)	4 (3–6)	5 (4–6)	3 (2.5–5)	0.005
Suprailiac skinfold thickness (mm)	4 (3–6)	5 (4–6)	3 (2.5–6)	0.012
**Maternal measurements**				
Pre-pregnacy maternal weight (kg)	67 ± 4.1	70 ± 4.3	64 ± 5.2	<0.001
Maternal weight at birth time (kg)	84.4 ± 12.2	89 ± 12	79 ± 9.9	<0.001
Maternal body mass index (kg/m^2^)	31.9 ± 4.3	33.5 ± 4.4	30.3 ± 3.6	0.001

**Table 2:** Comparison of cord blood results of groups.

	Total (n: 84)	İnfants of Diabetic Mothers (n: 42)	Control Group Infants (n: 42)	*p*
Perilipin-2 (ng/mL)	7.2 (5.0–13.6)	7.1 (5.3–11.8)	7.3 (5.0–18.4)	0.45
IGF-1 (μg/L)	56.5 (46.4–74.5)	56.8 (45–76)	56.5 (46–67)	0.97
IGF-2 (ng/mL)	190 (138–366)	204 (137–365)	182.7 (151–438)	0.43
Leptin (ng/mL)	8.0 (6.6–16.3)	8.9 (6.9–16)	8.0 (6.3–18)	0.97

**Table 3:** Correlation between anthropometric measurements and cord blood levels.

		Perilipin-2	IGF-1	IGF-2	Leptin
Birth weight Z-score	*p*	0.26	<0.001	0.29	0.11
	r	0.12	0.60	0.11	0.17
Birth height Z-score	*p*	0.16	0.014	0.52	0.04
	r	0.15	0.30	0.07	0.22
Head circumference Z-score	*p*	0.9	0.002	0.96	0.63
	r	0.014	0.38	0.005	0.052
Arm circumference	*p*	0.68	0.09	0.93	0.90
	r	0.044	0.20	−0.09	0.01
Triceps skinfold thickness	*p*	0.94	0.06	0.92	0.81
	r	0.007	0.22	−0.01	0.02
Biceps skinfold thickness	*p*	0.94	0.15	0.96	0.64
	r	0.008	0.17	−0.005	0.05
Subscapular skinfold thickness	*p*	0.48	0.11	0.98	0.71
	r	0.077	0.19	0.002	0.04
Suprailiac skinfold thickness	*p*	0.74	0.16	0.60	0.68
	r	0.036	0.17	−0.05	−0.04
Body mass index	*p*	0.50	<0.001	0.39	0.48
	r	0.074	0.49	0.094	0.077
Perilipin-2	*p*		0.07	<0.001	<0.001
	r		0.22	0.70	0.82

## Data Availability

Data are not stored publicly due to underage participants, but can be made available upon reasonable scientific request from the corresponding author.
